# ﻿*Cyrtandraobliquifolia* (Gesneriaceae), a new species from Kaua‘i, Hawaiian Islands

**DOI:** 10.3897/phytokeys.237.114704

**Published:** 2024-01-22

**Authors:** Kenneth R. Wood, Warren L. Wagner

**Affiliations:** 1 National Tropical Botanical Garden, 3530 Papalina Road, Kalāheo, HI 96741, USA National Tropical Botanical Garden Kalāheo United States of America; 2 Department of Botany, Smithsonian Institution, PO Box 37012, Washington, DC 20013-7012, USA Smithsonian Institution Washington, DC United States of America

**Keywords:** Conservation, critically endangered, *Cyrtandra*, Gesneriaceae, Hawaiian Islands, Kaua‘i

## Abstract

*Cyrtandraobliquifolia* K.R. Wood & W.L. Wagner (Gesneriaceae), a new shrub species known only from Kaua‘i, Hawaiian Islands, is described and illustrated with notes on its distribution, ecology, and conservation status. The new species is morphologically most similar to *Cyrtandrawawrae* C.B. Clarke but differs by its unique combination of oblique, non-peltate, auriculate leaf bases, more deeply divided calyx lobes, inflorescence with fewer flowers and lacking profusely umbellate cymes. *Cyrtandraobliquifolia* is known from only two localities which have undergone severe habitat degradation from landslides and invasive plants and animals and is determined to be Critically Endangered (CR) when evaluated under IUCN criteria.

## ﻿Introduction

*Cyrtandra* J.R. & G. Forster (Gesneriaceae) is composed of ca. 800 species that range across Southeast Asia and the Pacific (Atkins et al. 2013; Kleinkopf et al. 2019) including ca. 60 species occurring in the Hawaiian Islands, with ca. 50 being single-island endemics (SIE) (Wagner et al. 1999; Wagner and Roalson in prep.). On Kaua‘i, oldest of the high islands at ca. 4.7 million years (Price and Clague 2002), there are 14 species of *Cyrtandra*, including 13 SIE, three federally listed endangered, and one considered possibly extinct (i.e., *C.olona* C. Forbes), being last observed in 1909. Of the ca. 134 endemic vascular plants that are thought to be extinct throughout the Hawaiian Islands, five are in the genus *Cyrtandra* (Wood et al. 2019). The current status of all Hawaiian *Cyrtandra* is being investigated as part of ongoing studies by W. L. Wagner, E. Roalson, D. Brokaw, and several Hawaiian field botanists.

The continuing endangerment and loss of global biodiversity, especially in many insular ecosystems, has spurred botanists to rapidly assess and disseminate floristic data and conduct conservation collections to address potential extinctions. Over the last 30 years, ca. 28 new flowering plant and pteridophyte species have been discovered and described on Kaua‘i, in addition to about 29 taxa rediscovered after previously being considered possibly extinct. Familiarizing biologists with the distribution and abundance of these unique species is fundamental to our ability to conserve them. One pertinent example is the discovery and description of *Cyrtandrapaliku* W.L. Wagner, K.R. Wood & Lorence, an extremely narrow SIE species, restricted to a single cliff face on Kekoiki summit, northeastern Kaua‘i (Wagner et al. 2001), and now being monitored and conserved under the auspices of Hawaii’s Plant Extinction Prevention Program (PEPP).

**Figure 1.**
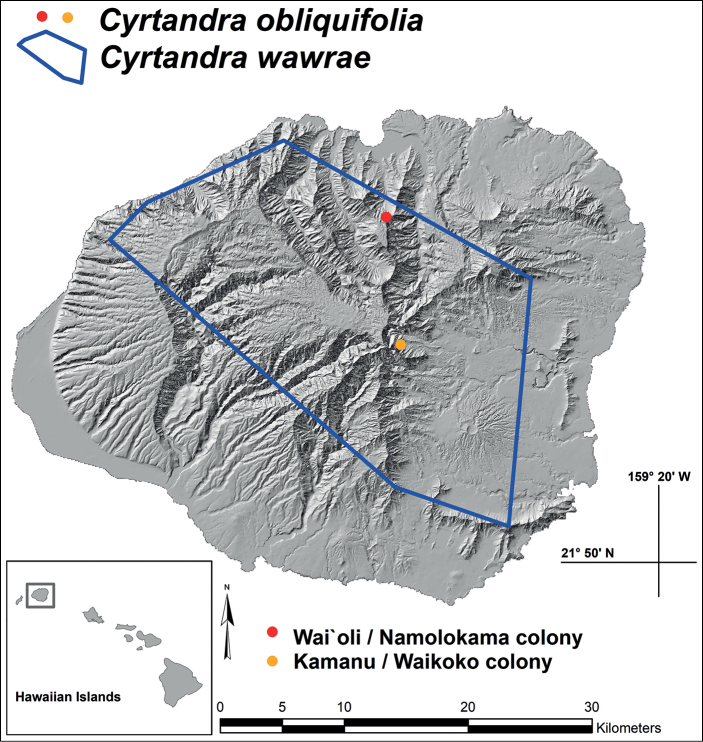
Distribution map (Kaua‘i, Hawaiian Islands) with dots representing the two known locations of *Cyrtandraobliquifolia* K.R. Wood & W.L. Wagner and polygon representing the broad distribution of *C.wawrae* C.B. Clarke.

In 2008 an unusual flowering specimen of *Cyrtandra* was collected by National Tropical Botanical Garden (**NTBG**) Science staff with laminae similar to *C.wawrae* C.B. Clarke in overall look and size but differed most notably in having non-peltate leaves. In this specimen the leaf blade base was asymmetrical on most of the leaves, and occasionally with symmetrical base, but not peltate. At the time it was referred to as an atypical *C.wawrae* and put aside for the time being. Further comparison showed additional morphological differences indicated in the diagnosis, most notably having only 3–5-flowered cymes instead of dense umbelliform cymes up to 17-flowered in *C.wawrae*. We included *C.obliquifolia* in ongoing molecular analyses and found it was distinctive yet closely allied to *C.wawrae* (Kleinkopf et al. 2019). During our investigation of this new species, we reexamined other collections in the PTBG and US herbaria and found that there was one additional collection made in 1993 from another locality in Wai‘oli Valley, on the northern slopes of Namolokama, over 10 km from the holotype location (Fig. 1).

## ﻿Methods

All measurements were taken from dried herbarium specimens, while overall plant size and population abundance were taken from field notes. For herbarium research we worked extensively with *Cyrtandra* specimens at BISH, PTBG, and US. The authors have examined all specimens cited in this paper. Measurements are presented in the description as follows: length × width, followed by units of measurements (mm or cm). We assessed the extinction risk for *Cyrtandraobliquifolia* following the IUCN Red List Categories and Criteria (IUCN 2012) and guidelines of the IUCN (2022). The extent of occurrence (EOO) and area of occupancy (AOO) were calculated by using ArcMap 10.6.1 (ESRI 2011) in relation to coordinates recorded while collecting both herbarium specimens.

## ﻿Taxonomic treatment

### ﻿ Cyrtandra obliquifolia

Taxon classificationPlantaeLamialesGesneriaceae

K.R.Wood & W.L.Wagner sp. nov.

F2B280E4-74DD-5C88-A412-3E450D28BAD8

urn:lsid:ipni.org:names:77334870-1

Figs 2 , 3

#### Diagnosis.

Morphologically, *Cyrtandraobliquifolia* is similar to *C.wawrae*, differing in having non-peltate leaves (*vs.* peltate), only 3–5-flowered cymes (*vs.* dense umbelliform cymes up to 17-flowered), corolla tube 10–11 mm long (*vs.* 13–17 mm long), and calyx ca. 10 mm long, the lobes lanceolate, 8–9.5 mm long, pilose within (*vs.* calyx 12–32 mm long, enclosing the fruit at maturity, the lobes deltate, 2–6(–10) mm long, glabrate to sparsely pilose).

#### Type.

**USA, Hawaiian Islands, Kaua‘i**: Līhu‘e District, below Kamanu Ridge, headwaters of Waikoko, 22.058641, -159.484138, 732 m, 12 Jan 2008, *Wood 12775* (holotype: PTBG1000002533!; isotype: BISH1152010!).

#### Description.

Small shrubs ca. 0.5–0.75 m tall; stems few-branched. ***Leaves*** opposite, those of a pair unequal, usually strongly asymmetrical, sometimes nearly symmetrical, coriaceous, very broadly ovate to broadly elliptic or sometimes suborbicular, 20–29 cm long, (7.8–)10–12.8 cm wide, upper surface sparsely to moderately pilose, the hairs with a broad base, lower surface densely velvety pilose, the hairs with a slightly broader base, sometimes gland-tipped, whitish to pale brown, margins dentate or serrate, moderately densely glandular pilose, apex acuminate, base asymmetrically cordate, auriculate, with one side extending 0.3–1.4 cm further than the other, sometimes cordate, petioles 7–13 cm long. ***Flowers*** 3–5 in cymes arising in the axils, glandular pilose throughout, peduncles stout, ca. 45 mm long, pedicels 15–25 mm long, bracts foliaceous, broadly ovate, ca. 20–25 mm long. ***Calyx*** nearly actinomorphic, ca. 10 mm long, the lobes lanceolate, 8–9.5 mm long, pilose, pilose within, apex acuminate, often slightly overlapping near base. ***Corolla*** white, tube cylindrical, 8.5–15 mm long, pilose, upper lobes reniform, ca. 3 mm long, ca. 4 mm wide, lower lobes rhombic-ovate, 3–4 mm long, 6–8 mm wide; ovary glabrous; style 5–10 mm long, glabrous. ***Berries*** not seen. ***Seeds*** not seen.

#### Additional specimen examined.

**USA, Hawaiian Islands, Kaua‘i**: Hanalei District, Wai‘oli Valley, slopes of Namolokama, hanging valley east of main waterfall, 22.151496, -159.495704, 835 m, 21 Jan 1993, *Perlman, Lorence, Flynn & Wood 13259* (PTBG, US).

#### Phenology.

*Cyrtandraobliquifolia* has been observed with flower during the month of January.

#### Etymology.

The species epithet is from the Latin *obliquus* meaning slanting or unequal sides, and *folius* for leaf.

#### Affinities.

**Figure 2.**
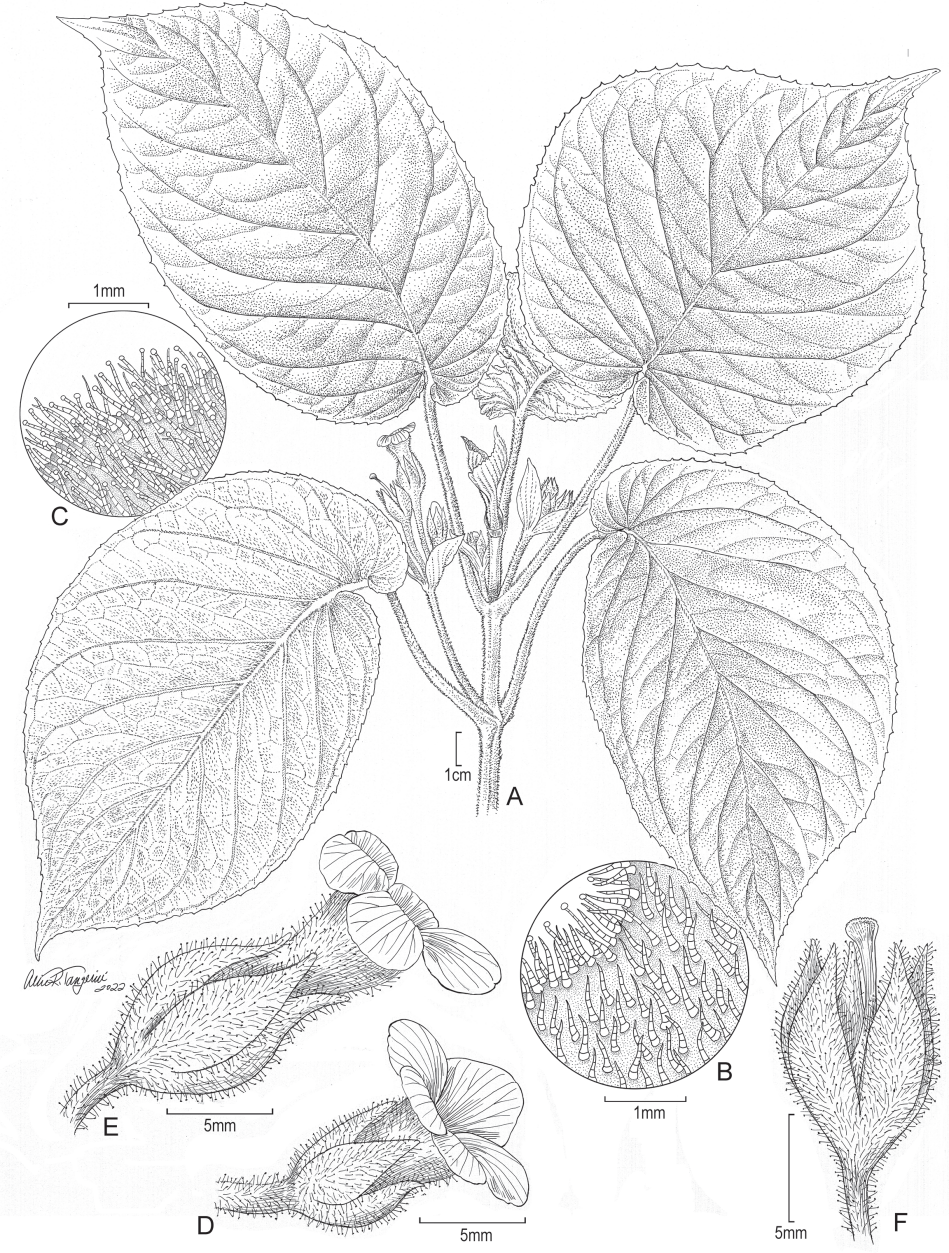
*Cyrtandraobliquifolia* K.R. Wood & W.L. Wagner **A** habit **B** pubescence on upper leaf surface and margin **C** pubescence on lower surface and margin **D** flower in early anthesis (male phase) **E** flower in full anthesis (female stage) **F** calyx after flowering showing stigma. Drawn from holotype and augmented with photograph of plant that holotype was taken from (**A–D, F**) and from close-up photo of plant that holotype was taken from (**E**).

**Figure 3.**
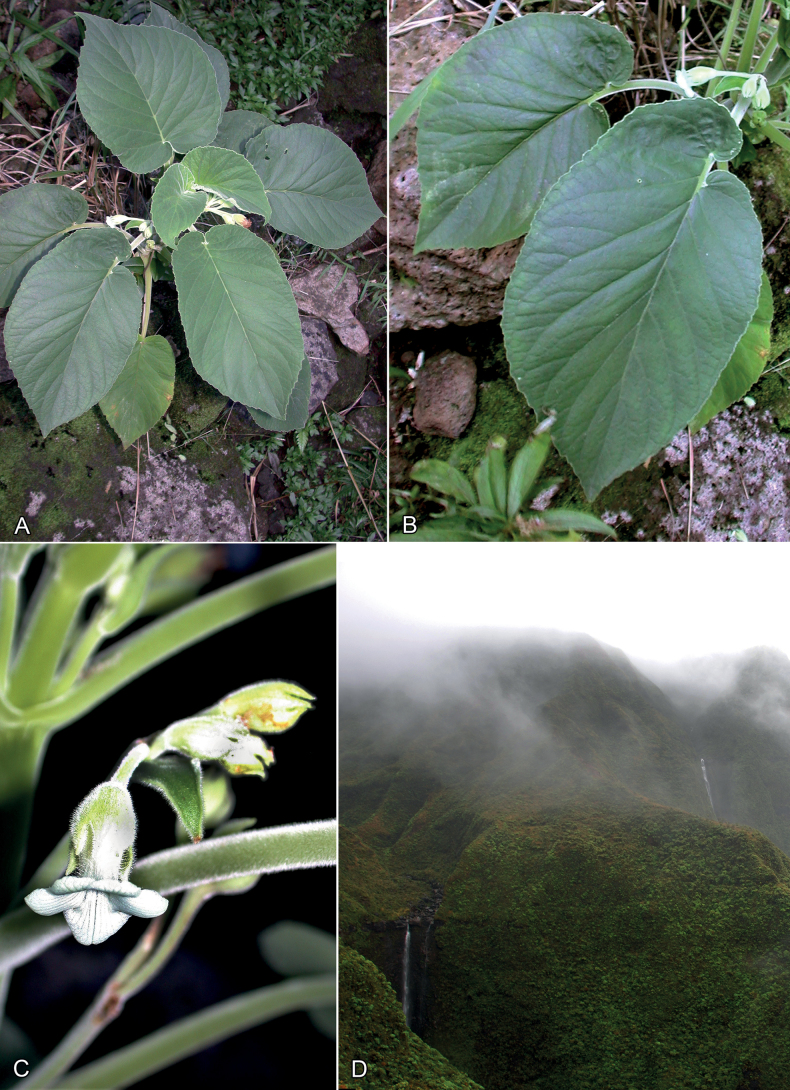
*Cyrtandraobliquifolia* K.R. Wood & W.L. Wagner from headwaters of Waikoko Valley where holotype was collected **A, B** habit **C** close-up of early anthesis flower **D** general habitat of type locality. All photos by K.R. Wood.

**Figure 4.**
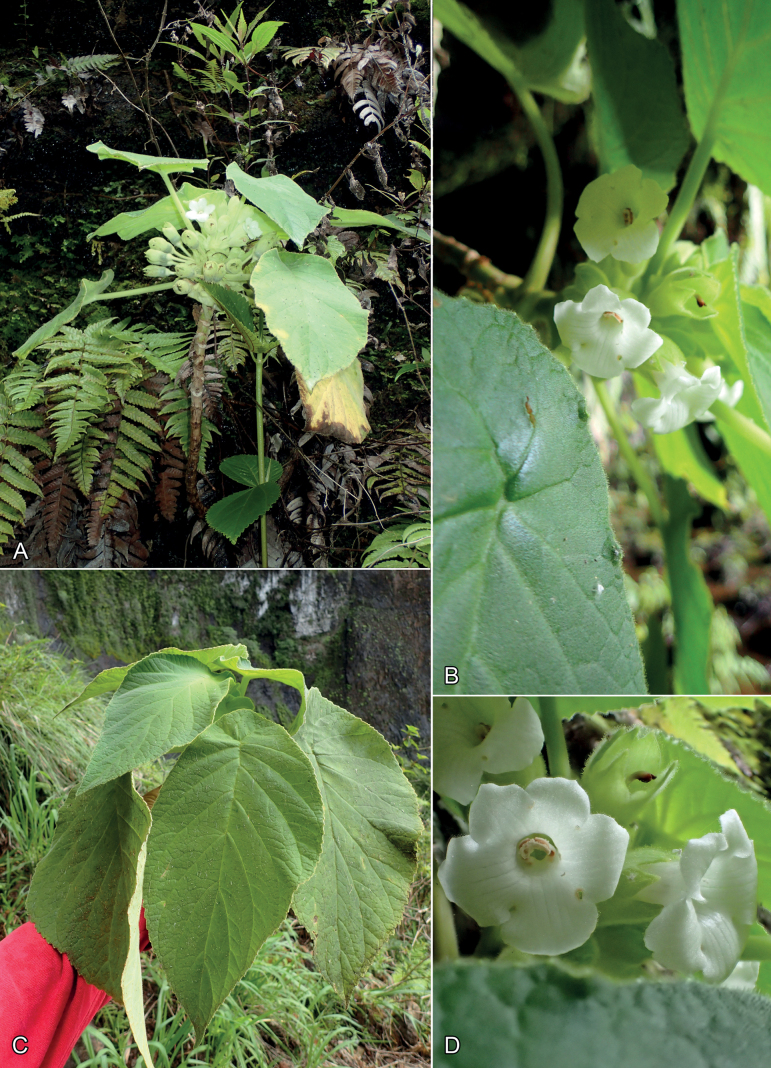
*Cyrtandrawawrae* C.B. Clarke **A** habit with inflorescence **B** close-up of inflorescence and peltate leaf **C** peltate leaves **D** close-up of corolla and side view of calyx. All photos by K.R. Wood **A** 17 June 2017, Wailua **B** Wailua, *Wood 17317* (PTBG) **C, D** Lumahai, *Wood 17398* (PTBG).

**Figure 5.**
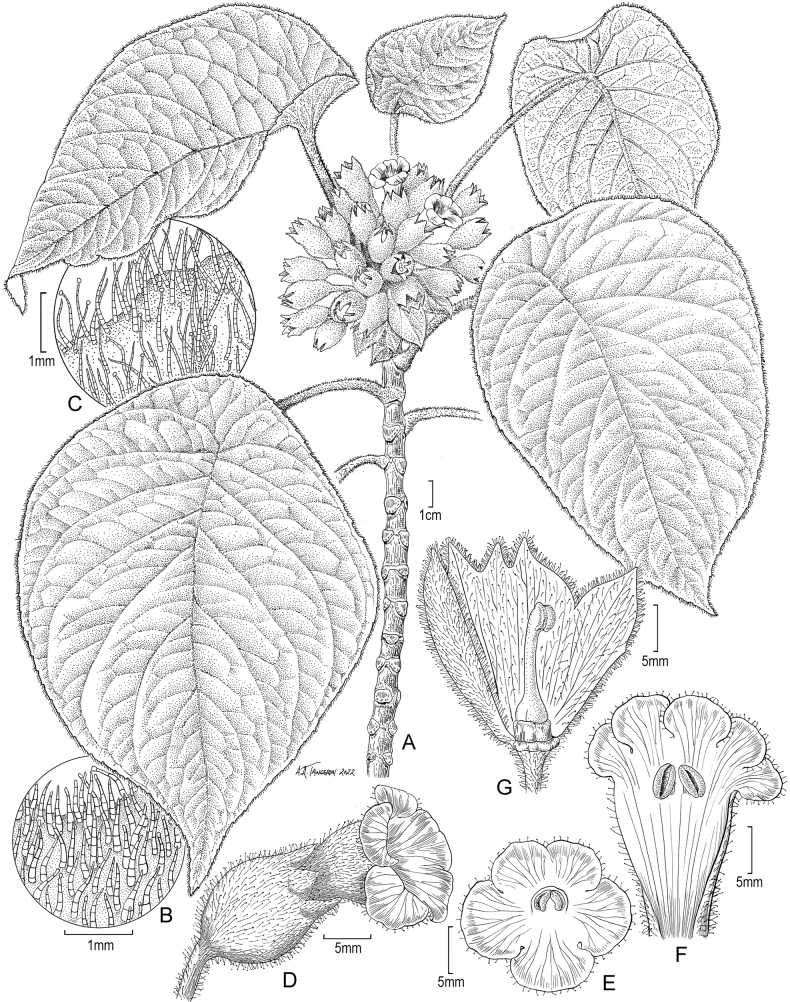
*Cyrtandrawawrae* C.B. Clarke **A** habit showing dense umbelliform cymes **B** pubescence on upper surface and margin **C** pubescence on lower surface and margin **D** flower, lateral view, in full anthesis (female stage) **E** face view of corolla **F** corolla longitudinal section showing inserted stamens **G** calyx longitudinal section after flowering showing stigma. Drawn from *Wood 907* (US), Kalalau Rim (**A–D, F, G**), and photo of *Wood 17398*, upper Lumahai (**E**). Illustration augmented with photos of habit (*Wood 17317*) and leaves from (*Perlman & Wood 13599*) (A), and photos of flowers (*Wood 17398*) (**D**).

*Cyrtandraobliquifolia* is closely related to *C.wawrae* as shown by the similar morphology when first collected. A sample of it was included as one of the 31 samples in a hyb-seq phylogenomic analysis of the Hawaiian lineage and was strongly supported as sister to *C.wawrae* and the pair an early-diverging one in the Hawaiian lineage (Kleinkopf et al. 2019). Our morphological comparisons indicated there are a number of other differences between these two species (Figs 2–5; Table 1).

**Table 1.** Comparison of morphological and ecological characters of two similar Kaua‘i species of Hawaiian *Cyrtandra*.

Character	*C.wawrae*	*C.obliquifolia*
Leaves	Peltate, light green, petioles inserted 1–5.5 cm from base, the base rounded to truncate, occasionally broadly cuneate	Not peltate, dark green, the base asymmetrically cordate, with one side extending 0.3–1.4 cm further than the other, sometimes cordate
Inflorescence	with 6–17 flowers, in dense umbelliform cymes arising in the axils just below the current leaves, peduncles 1–35 mm long	with 3–5 flowers in cymes arising in the axils, peduncles 45 mm long
Calyx	12–32 mm long, enclosing the fruit at maturity, the lobes deltate, 2–6(–10) mm long, glabrate to sparsely pilose within	ca. 10 mm long, the lobes lanceolate, 8–9.5 mm long, pilose within
Corolla tube	13–17 mm long	8.5–15 mm long
Habitat	Mesic to wet forest	Wet forest

#### Distribution and ecology.

*Cyrtandraobliquifolia* is endemic to the volcanic island of Kaua‘i where there is uncertainty whether any surviving plants remain. The oldest of the main Hawaiian Islands and summiting at 1598 m, Kaua‘i contains the highest level of floristic diversity throughout the archipelago with ca. 250 SIE and a total of ca. 673 native vascular plant taxa (Wagner et al. 1999; Vernon and Ranker 2013; Wood et al. 2016; Rønsted et al. 2022). Local botanists estimate around 21 of those Kaua‘i SIE taxa are possibly extinct with no known wild individuals (Wood et al. 2019). Kaua‘i features a highly variable physical geography compared to the younger high Hawaiian Islands, exemplified by deeply eroded and isolated drainages, well-defined canyons, tall coastal seacliffs, along with lowland and montane bogs and interior wet cliff habitats. Although much of the lowland dry habitats had been altered or lost by the time the first European explorer, Captain Cook, made contact on Kaua‘i in 1778, there still remains a fair abundance of mesic and wet forest ecosystems, much of which are extremely rugged and unexplored. It was around Kaua‘i’s saturated cliffs and towering waterfalls that *Cyrtandraobliquifolia* was documented.

A vegetative plant of *Cyrtandraobliquifolia* was first documented in 1993 and noted to be occasional around an inaccessible hanging valley above Wai`oli Stream, Kaua‘i, on the isolated northern face of Namolokama Mountain at 835 m elev. (Fig. 1). This survey was facilitated by helicopter. Few botanists have since returned to this exact location, and although no individuals have since been reported around this site, further surveys are recommended along the wet cliffs and surrounding forests of Namolokama’s isolated hanging valleys. It should be noted that several rare plant taxa in this area have declined as a result of an influx of weedy invasive plants establishing themselves post-hurricane Iniki (ca. 1992). In Aug 2023, the first author flew into the Wai`oli region and visited the area just below the hanging valley where *Perlman 13259* was collected, and, unfortunately, confirms the serious degradation of the region since the 1990s. A second colony of *C.obliquifolia* was documented in flower 15 years later (i.e., Jan 2008) along the windward headwater drainage of Waikoko Stream at 732 m elev. (east central Kaua‘i; Fig. 1). After discovery, this region suffered a devastating landslide which possibly destroyed the known colony of ca. 20 individuals of *C.obliquifolia*, along with the last known Kaua‘i colony of *Lysimachiafilifolia* C.N. Forbes & Lydgate (Primulaceae). Still, there is extensive habitat surrounding the Waikoko headwaters where additional colonies of *C.obliquifolia* could still occur (Figs 1, 3D).

The plant community where both colonies of *Cyrtandraobliquifolia* were found is a *Metrosideros* Banks ex Gaertn. (Myrtaceae) / *Cheirodendron* Nutt. ex Seem. (Araliaceae) lowland wet forest. These forests are low statured and partially open where they flourish around the bases of seeping vertical basalt wet cliff communities. Associate plant species in the area include a rich mix of endemic native sedges, grasses, ferns, herbs, shrubs, and stunted trees, many of the species being unique single-island endemics. Wood and Knope (2023) defined the general ecology of these wet forests and cliffs in their publication of *Bidenswailele* K.R. Wood & Knope, a recently described endemic perennial herb also documented around the holotype area of *C.obliquifolia*. From our observations, *C.obliquifolia* inhabits the open banks of streams within these lowland wet forests, in addition to the lower walls of the surrounding wet cliff community. Associated genera of trees in the type locality include *Polyscias* J.R. Forst. & G. Forst. (Araliaceae); *Pritchardia* Seem. & H. Wendl. (Arecaceae); *Dubautia* Gaudich. (Asteraceae); *Cyanea* Gaudich., *Lobelia* Plum. ex L. (Campanulaceae); *Perrottetia* Kunth (Dipentodontaceae); *Antidesma* L., *Euphorbia* L. (Euphorbiaceae); *Hydrangea* Gronov. (Hydrangeaceae); *Syzygium* Gaertn. (Myrtaceae); *Bobea* Gaudich., *Coprosma* J.R. Forst. & G. Forst., *Kadua* Cham. & Schltdl. (some being smaller shrubs), *Psychotria* L. (Rubiaceae); *Melicope* J.R. Forst. & G. Forst. (Rutaceae), and *Pipturus* Wedd., *Touchardia* Gaudich., *Urera* Gaudich. (Urticaceae). Genera of sedges and grasses include *Carex* L., *Cyperus* L., *Machaerina* Vahl (Cyperaceae); *Eragrostis* Wolf, *Isachne* R. Br. (Poaceae); ferns of *Asplenium* L., *Hymenasplenium* Hayata (Aspleniaceae); *Deparia* Hook. & Grev., *Diplazium* Sw. (Ath**y**riaceae); *Sadleria* Kaulf. (Blechnaceae); *Cibotium* Kaulf. (Cibotiaceae); *Microlepia* C. Presl (Dennstaedtiaceae); *Hoiokula* S.E. Fawc. & A.R. Sm., *Menisciopsis* (Holttum) S.E. Fawc. & A.R. Sm. (Thelypteridaceae); herbs and shrubs include *Bidens* L. (Asteraceae); *Cyrtandra* J.R. Forst. & G. Forst. (Gesneriaceae); *Gunnera* L. (Gunneraceae); *Plantago* L. (Plantaginaceae); *Lysimachia* Tourn. ex L. (Primulaceae); and the woody climber *Freycinetia* Gaudich. (Pandanaceae).

#### Preliminary conservation assessment.

***IUCN Red List Category*.** When evaluated using the World Conservation Union (IUCN 2012, 2022) guidelines and criteria for endangerment, *Cyrtandraobliquifolia* falls into the Critically Endangered (CR) category. Our evaluation following the IUCN hierarchical alphanumeric numbering system of criteria and conditions is CR B1ab(i,ii,iii,iv,v); B2ab(i,ii,iii,iv,v); C2a(i); D; which reflects a severely limited EOO of less than 100 km^2^ (i.e., 4 km^2^) an AOO of less than 10 km^2^ (i.e., 1 km^2^), a severely fragmented distribution with two subpopulations separated by 10 km, a continued decline in quality of habitat inferred, and a population of ca. 40 mature plants observed, ranging between 732 and 835 m elev. The continued decline in quality of habitat for *Cyrtandraobliquifolia* is evidenced by severe habitat degradation from invasive plants and animals, in addition to hurricane force winds, flash floods and landslides (especially after torrential rains). Destructive non-native animals in the area include (*Susscrofa* L.), and rats (*Rattus* spp.), along with introduced slugs and insects. Specific invasive non-native plants include *Ageratumconyzoides* L., *Erigeronbonariensis* L., *E.karvinskianus* DC., (Asteraceae); *Buddlejaasiatica* Lour. (Buddlejaceae); *Sphaeropteriscooperi* (Hook. ex F. Muell.) R.M. Tryon (Cyatheaceae); *Juncusplanifolius* R. Br. (Juncaceae); *Miconiacrenata* (Vahl.) Michelang. (Melastomataceae); *Psidiumcattleyanum* Sabine (Myrtaceae); *Andropogonglomeratus* (Walter) Britton, Sterns & Poggenb., *Axonopusfissifolius* (Raddi) Kuhlm., *Sacciolepisindica* (L.) Chase (Poaceae); *Adiantumraddianum* C. Presl (Pteridaceae); *Rubusrosifolius* Sm. (Rosaceae); and *Hedychiumgardnerianum* Sheph. ex Ker Gawl. (Zingiberaceae).

As exhaustive surveys have not yet been conducted in the surrounding habitats where *Cyrtandraobliquifolia* has been documented, we do not believe this species is extinct in the wild.

We are hoping that this publication with description and illustrations will give botanists incentive and guidance to look for additional colonies of this beautiful gesneriad around the hanging valleys that surround Namolokama, along with searches along the back walls of Waikoko drainage and wet cliffs of Kamanu ridge which rise up to the very summit of Kawaikini, Kaua‘i. We also recommend concerted inventories be initiated deep into the great central, headwater drainage of Olokele, which is quite near the holotype region yet privately owned and in need of special permitting for exploration.

## Supplementary Material

XML Treatment for Cyrtandra obliquifolia

## ﻿Additional information

### Author ORCIDs

Kenneth R. Wood https://orcid.org/0000-0001-6446-1154

Warren L. Wagner https://orcid.org/0000-0001-5012-8422

### Data availability

All of the data that support the findings of this study are available in the main text.

## References

[B1] Atkins HJ Bramley GLC Clark JR (2013) The taxonomy of *Cyrtandra* (Gesneriaceae): Current knowledge, future directions. Selbyana 31: 65–253. https://journals.flvc.org/selbyana/article/view/123020

[B2] ESRI (2011) ArcGIS Desktop: Release 10.6.1. Environmental Systems Research Institute, Redlands, CA.

[B3] IUCN (2012) IUCN Red List Categories and Criteria Version 3.1 (2^nd^ edn.). Prepared by the IUCN Criteria Review Working Group. IUCN, Cambridge.

[B4] IUCN (2022) Guidelines for using the IUCN Red List Categories and Criteria. Version 15.1. Prepared by the Standards and Petitions Committee. https://www.iucnredlist.org/documents/RedListGuidelines.pdf [Accessed 17.12.2023]

[B5] Kleinkopf J Roberts W Wagner WL Roalson EH (2019) Diversification of Hawaiian *Cyrtandra* (Gesneriaceae) under the influence of incomplete lineage sorting and hybridization. Journal of Systematics and Evolution 57(6): 561–578. 10.1111/jse.12519

[B6] Price JP Clague DA (2002) How old is the Hawaiian biota? Geology and phylogeny suggest recent divergence. Proceedings of the Royal Society B, Biological Sciences 269(1508): 2429–2435. 10.1098/rspb.2002.2175 PMC1691179 12495485

[B7] Rønsted N Walsh SK Clark M Edmonds M Flynn T Heintzman S Loomis A Lorence D Nagendra U Nyberg B Opgenorth M Weisenberger L Williams A Wolkis D Wood KR Keir M (2022) Extinction risk of endemic vascular flora of Kaua’i Island, Hawai’i, based on IUCN assessments. Conservation Biology 36(4): e13896. 10.1111/cobi.13896 PMC9544520 35146804

[B8] Vernon AL Ranker TA (2013) Current status of the ferns and lycophytes of the Hawaiian Islands. American Fern Journal 103(2): 59–111. 10.1640/0002-8444-103.2.59

[B9] Wagner WL Herbst DR Sohmer SH (1999) Manual of the Flowering Plants of Hawai‘i, revised edition with supplement by Wagner WL and Herbst DR. University of Hawai‘i Press (2 Vols). Bishop Museum Special Publication 97, 1919 pp.

[B10] Wagner WL Wood KR Lorence DH (2001) A new species of *Cyrtandra* (Gesneriaceae) from Kaua’i, Hawaiian Islands. Novon 11(1): 146–152. 10.2307/3393224

[B11] Wood KR Knope ML (2023) *Bidenswailele* (Asteraceae, Coreopsideae): A new critically endangered species from Kaua‘i, Hawaiian Islands. International Journal of Plant Sciences 184(5): 378–386. 10.1086/724311

[B12] Wood KR Lorence DH Kiehn M (2016) *Coprosmakawaikiniensis* (Rubiaceae) a new species from the *Dubautia*-*Sadleria* shrubland-fernland community on Kaua’i, Hawaiian Islands. PhytoKeys 60: 21–32. 10.3897/phytokeys.60.6406 PMC4816988 27081342

[B13] Wood KR Oppenheimer H Keir M (2019) A checklist of endemic Hawaiian vascular plant taxa from the Hawaiian Islands that are considered possibly extinct in the wild. Technical Report 314. The National Tropical Botanical Garden.

